# Extensive loss of Wnt genes in Tardigrada

**DOI:** 10.1186/s12862-021-01954-y

**Published:** 2021-12-27

**Authors:** Raul A. Chavarria, Mandy Game, Briana Arbelaez, Chloe Ramnarine, Zachary K. Snow, Frank W. Smith

**Affiliations:** Biology Department, University of North Florida, Jacksonville, FL USA

**Keywords:** Wnt signaling, Tardigrada, Miniaturization, Posterior growth, Panarthropoda

## Abstract

**Background:**

Wnt genes code for ligands that activate signaling pathways during development in Metazoa. Through the canonical Wnt (cWnt) signaling pathway, these genes regulate important processes in bilaterian development, such as establishing the anteroposterior axis and posterior growth. In Arthropoda, Wnt ligands also regulate segment polarity, and outgrowth and patterning of developing appendages. Arthropods are part of a lineage called Panarthropoda that includes Onychophora and Tardigrada. Previous studies revealed potential roles of Wnt genes in regulating posterior growth, segment polarity, and growth and patterning of legs in Onychophora. Unlike most other panarthropods, tardigrades lack posterior growth, but retain segmentation and appendages. Here, we investigated Wnt genes in tardigrades to gain insight into potential roles that these genes play during development of the highly compact and miniaturized tardigrade body plan.

**Results:**

We analyzed published genomes for two representatives of Tardigrada, *Hypsibius exemplaris* and *Ramazzottius varieornatus*. We identified single orthologs of *Wnt4*, *Wnt5*, *Wnt9*, *Wnt11*, and *WntA*, as well as two *Wnt16* paralogs in both tardigrade genomes. We only found a *Wnt2* ortholog in *H. exemplaris*. We could not identify orthologs of *Wnt1*, *Wnt6*, *Wnt7*, *Wnt8*, or *Wnt10*. We identified most other components of cWnt signaling in both tardigrade genomes. However, we were unable to identify an ortholog of *arrow*/*Lrp5/6*, a gene that codes for a Frizzled co-receptor of Wnt ligands. Additionally, we found that some other animals that have lost several Wnt genes and are secondarily miniaturized, like tardigrades, are also missing an ortholog of *arrow*/*Lrp5/6*. We analyzed the embryonic expression patterns of Wnt genes in *H. exemplaris* during developmental stages that span the establishment of the AP axis through segmentation and leg development. We detected expression of all Wnt genes in *H. exemplaris* besides one of the *Wnt16* paralogs. During embryo elongation, expression of several Wnt genes was restricted to the posterior pole or a region between the anterior and posterior poles. Wnt genes were expressed in distinct patterns during segmentation and development of legs in *H. exemplaris*, rather than in broadly overlapping patterns.

**Conclusions:**

Our results indicate that Wnt signaling has been highly modified in Tardigrada. While most components of cWnt signaling are conserved in tardigrades, we conclude that tardigrades have lost *Wnt1*, *Wnt6*, *Wnt7*, *Wnt8*, and *Wnt10*, along with *arrow*/*Lrp5/6*. Our expression data may indicate a conserved role of Wnt genes in specifying posterior identities during establishment of the AP axis. However, the loss of several Wnt genes and the distinct expression patterns of Wnt genes during segmentation and leg development may indicate that combinatorial interactions among Wnt genes are less important during tardigrade development compared to many other animals. Based on our results, and comparisons to previous studies, we speculate that the loss of several Wnt genes in Tardigrada may be related to a reduced number of cells and simplified development that accompanied miniaturization and anatomical simplification in this lineage.

**Supplementary Information:**

The online version contains supplementary material available at 10.1186/s12862-021-01954-y.

## Background

Wnt genes are a group of paralogous ligand-coding genes that play instrumental roles in regulating animal development through both canonical and non-canonical Wnt signaling pathways [1–6]. One important role of Wnt genes is regulating the development of primary body axes [7–14]. In Bilateria, the anteroposterior (AP) body axis is the primary body axis. In many bilaterians, polarized expression of Wnt genes establishes the AP axis by promoting posterior identity and suppressing anterior identity [9, 15–19]. After establishing the AP axis in many bilaterians, canonical Wnt (cWnt) signaling then regulates posterior growth [20–29]. Establishing the AP axis and regulating posterior growth are most likely ancestral functions of Wnt genes in Bilateria [9, 20]. Therefore, Wnt genes may have played a key role in the origin of the AP axis of bilaterians [30].

Wnt genes also regulate development of body plan characteristics at more narrow taxonomic scales within Bilateria. For example, studies of the *Wnt1* ortholog *wingless* (*wg*) have played a key part in deciphering the development of the segmented body plans of the hyperdiverse Arthropoda. In *Drosophila melanogaster*, *wg* participates in the segment polarity network with *engrailed* (*en*) *hedgehog* (*hh*), and other genes [31–34]. This network is required for segment formation and intrasegmental patterning in arthropods. Later, *wg* initiates growth of the proximodistal (PD) axis in legs and other appendage types and then specifies ventral fate in these appendages [35–42]. These functions of *wg* are highly conserved across Arthropoda, although *wg* most likely does not function as a segment polarity gene in spiders [35, 43–58]. In arthropods, other Wnt genes are expressed in patterns that resemble *wg* expression, indicating that several Wnt genes may be acting redundantly or combinatorially to regulate development in this lineage [3, 58–63]. Wnt genes have also been studied in two species of Onychophora [28, 64–66], the likely sister group of Arthropoda [67–69]. The segment polarity network is most likely conserved in onychophorans, in which it may regulate intrasegmental patterning, but is unlikely to play a role in segment formation [64–66, 70]. Additionally, Wnt genes appear to play roles in the segmentation process and during appendage development in onychophorans that are not characteristic of arthropods [28].

Several lines of evidence suggest that Tardigrada represents the outgroup of an arthropod + onychophoran lineage in a monophyletic Panarthropoda [67, 68, 71], although other relationships have been recovered in some analyses [69, 72], making studies of tardigrades critical for resolving where in panarthropod phylogeny important roles of Wnt genes evolved. Additionally, the unique body plan and developmental mode of Tardigrada raises intriguing questions about the roles of Wnt genes in this lineage. Tardigrades have a highly compact body plan composed of a single-segment head and four leg bearing trunk segments. This compact body plan evolved in conjunction with miniaturization [73, 74]. Embryonic expression analyses of Hox genes and other AP axis patterning genes in the tardigrade *Hypsibius exemplaris* revealed that tardigrades have lost a mid-trunk region [75, 76]. The mid-trunk region that is missing in tardigrades develops by posterior growth in other panarthropods [75, 77]. Tardigrades have lost posterior growth; all segments develop nearly simultaneously in these animals [78–81]. Additionally, the proximodistal (PD) axis of *H. exemplaris* legs is missing an intermediate domain defined by *dachshund* expression that is found in onychophorans, arthropods, and other animals [82]. The fact that Wnt genes regulate the development of both the AP axis and PD axis in other animals suggests that the evolution of the compact tardigrade body plan may be associated with modifications to the functions of Wnt genes in Tardigrada.

Here, we present the first study of Wnt genes in Tardigrada. We discovered that several Wnt genes and *arrow*, an ortholog of *Lrp5* and *Lrp6* in vertebrates, which codes for a co-receptor of Wnt ligands, have been lost in the tardigrade lineage. Based on comparisons to other metazoan genomes, it appears that the loss of several Wnt genes and *arrow*/*Lrp5/6* are common features of genome evolution in secondarily miniaturized animals, like tardigrades. Expression patterns of Wnt genes in *H. exemplaris* embryos suggest that these genes play roles during establishment of the AP axis, segmentation, endomesodermal development, foregut development, and leg development. Interestingly, Wnt genes exhibit distinct expression patterns during segmentation and leg development, rather than similar expression patterns like in many other animals. This fact, along with the extensive loss of Wnt genes in Tardigrada, may indicate that tardigrade Wnt genes exhibit reduced combinatorial interactions compared to some other animals. We suggest that the extensive loss of Wnt genes in Tardigrada is associated with miniaturization and the associated reduction of cell number and simplified developmental mechanisms that accompany this process.

## Results

### Phylogenetic and genomic analyses of tardigrade Wnt genes

**Fig. 1:**
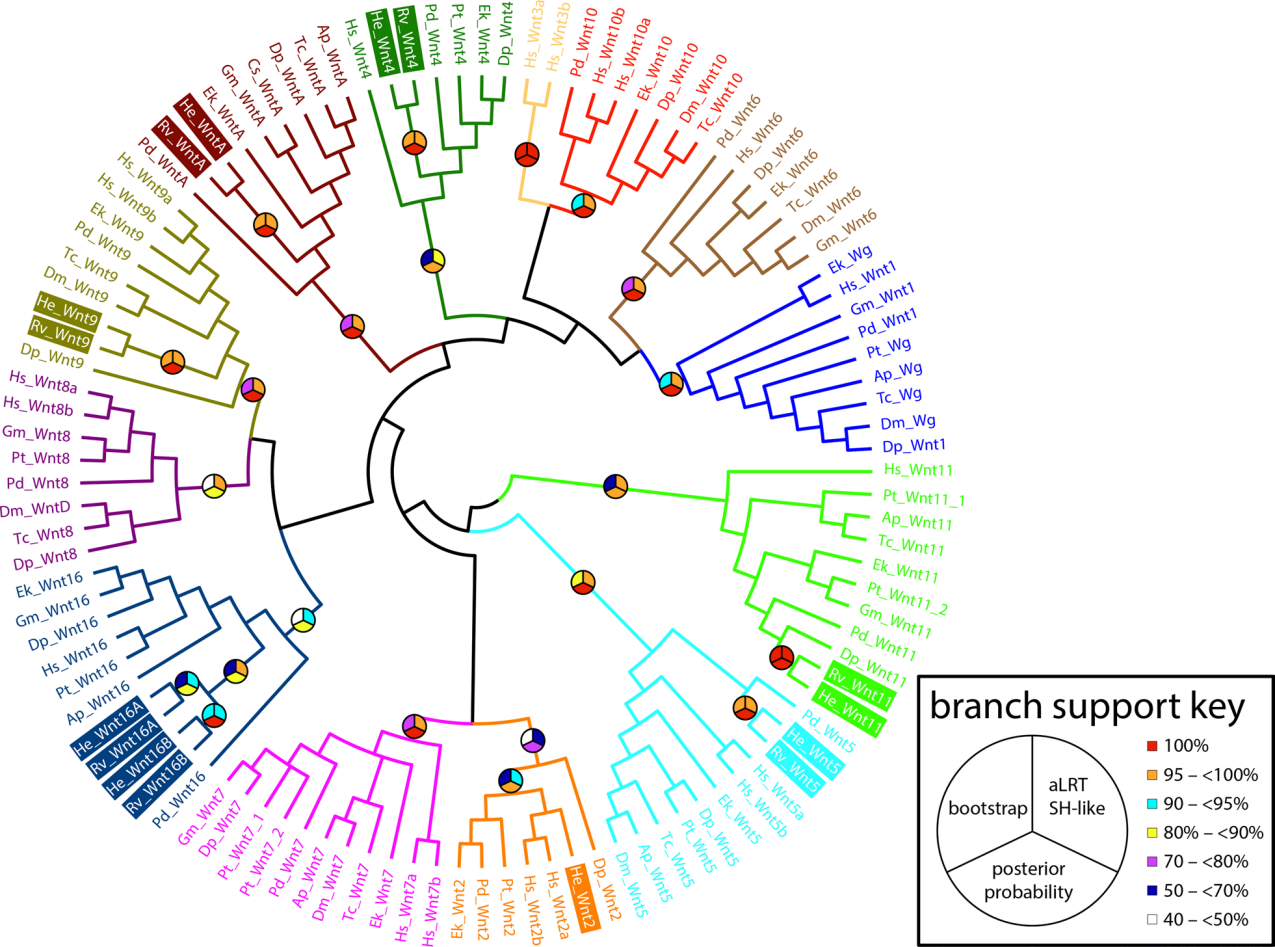
Majority rule consensus tree of Wnt ligands. For the sake of space, taxon abbreviations are used, and branch lengths are not diagrammed. See Additional file 1: Fig. S1 for a version of this tree that includes branch length information. For simplicity, only branch support values relevant to determining the identity of the candidate tardigrade Wnt ligands are shown. Tardigrade sequences are in colored boxes. Bootstrap supports are shown as percentages out of 500 replicates. Species abbreviations: Ap, *Acyrthosiphon pisum*; Cs, *Cupiennius salei*; Dp, *Daphnia pulex*; Dm, *Drosophila melanogaster*; Ek, *Euperipatoides kanangrensis*; Gm, *Glomeris marginata*; He, *Hypsibius exemplaris*; Hs, *Homo sapiens*; Is, *Ixodes scapularis*; Pd, *Platynereis dumerilii*; Pt, *Parasteatoda tepidariorum*; Rv, *Ramazzottius varieornatus*; Tc, *Tribolium castaneum*

We identified eight candidate Wnt genes in the *H. exemplaris* genome and seven Wnt genes in the genome of *R. varieornatus* by BLAST search. We confirmed that all candidate Wnt genes encode a Wnt superfamily domain by CD-search analysis [83]. Our phylogenetic analyses revealed that both *H. exemplaris* and *R. varieornatus* possess orthologs of *Wnt4*, *Wnt5*, *Wnt9*, *Wnt11*, *Wnt16*, and *WntA* (Fig. 1; Table 1; Additional file 1: Fig. S1). These species each had two paralogs of *Wnt16*, which we refer to as *Wnt16A* and *Wnt16B*. Additionally, *H. exemplaris* possesses an ortholog of *Wnt2*. We did not detect orthologs of *Wnt1*, *Wnt6*, *Wnt7*, *Wnt8*, and *Wnt10*—Wnt genes that would be predicted to be present in tardigrade genomes [2, 3, 5, 6, 84]. Next, we investigated the arrangement of Wnt genes in the genomes of *H. exemplaris* and *R. varieornatus* [71, 85]. In *H. exemplaris*, all Wnt genes were found on unique scaffolds (Table 1). Scaffolds with Wnt genes ranged in length from 95,696 to 1,074,739 nt in this species. In *R. varieornatus*, scaffolds with Wnt genes ranged in length from 1,744,794–9,333,084 nt. Only *Wnt9* and *WntA* were found on the same scaffold, but these genes were 829,930 nt apart on this scaffold.

**Table 1:** Canonical Wnt signaling components in tardigrades. GenBank accession numbers and scaffold numbers are from previously published genome studies [71, 85]. An ortholog of Arrow was not found

Function	Ortholog	Protein accession numbers		Scaffold numbers	
		*H. exemplaris*	*R. varieornatus*	*H. exemplaris*	*R. varieornatus*
Ligand	Wnt2	OWA52741.1	–	scaffold0284	–
	Wnt4	OQV20568.1	GAV00263.1	scaffold0029	BDGG01000006
	Wnt5	OQV25062.1	GAU92975.1	scaffold0004	BDGG01000002
	Wnt9	OQV11710.1	GAU97803.1	scaffold0163	BDGG01000004
	Wnt11	OQV21261.1	GAU87525.1	scaffold0024	BDGG01000001
	Wnt16A	OQV19782.1	GAU94914.1	scaffold0035	BDGG01000003
	Wnt16B	OQV22138.1	GAV05665.1	scaffold0019	BDGG01000012
	WntA	OQV17790.1	GAU98124.1	scaffold0056	BDGG01000004
Transmembrane transport	Wntless	OQV19301.1	GAV08953.1	scaffold0041	BDGG01000019
Receptor	Fz1	OQV23182.1	GAU93634.1	scaffold0013	BDGG01000002
	Fz2	OQV23168.1	GAU93659.1	scaffold0013	BDGG01000002
	Fz3	OQV21307.1	GAU87444.1	scaffold0024	BDGG01000001
	Fz4	OQV18791.1	GAV00601.1	scaffold0046	BDGG01000006
	Arrow	–	–	–	–
Signal transduction	Dishevelled	OQV23134.1	GAV07421.1	scaffold0013	BDGG01000015
	Armadillo	OWA50075.1	GAV07811.1	scaffold0181	BDGG01000016
Transcription factor	Pangolin	OQV17172.1	GAV07782.1	scaffold0065	BDGG01000016
cWnt inhibition	Shaggy	OQV18828.1	GAU89015.1	scaffold0045	BDGG01000001
	APC	OQV22882.1	GAV03262.1	scaffold0015	BDGG01000009
	Axin	OQV22259.1	GAV03998.1	scaffold0018	BDGG01000010

### Identification of conserved components of canonical Wnt signaling in Tardigrada

**Fig. 2:**
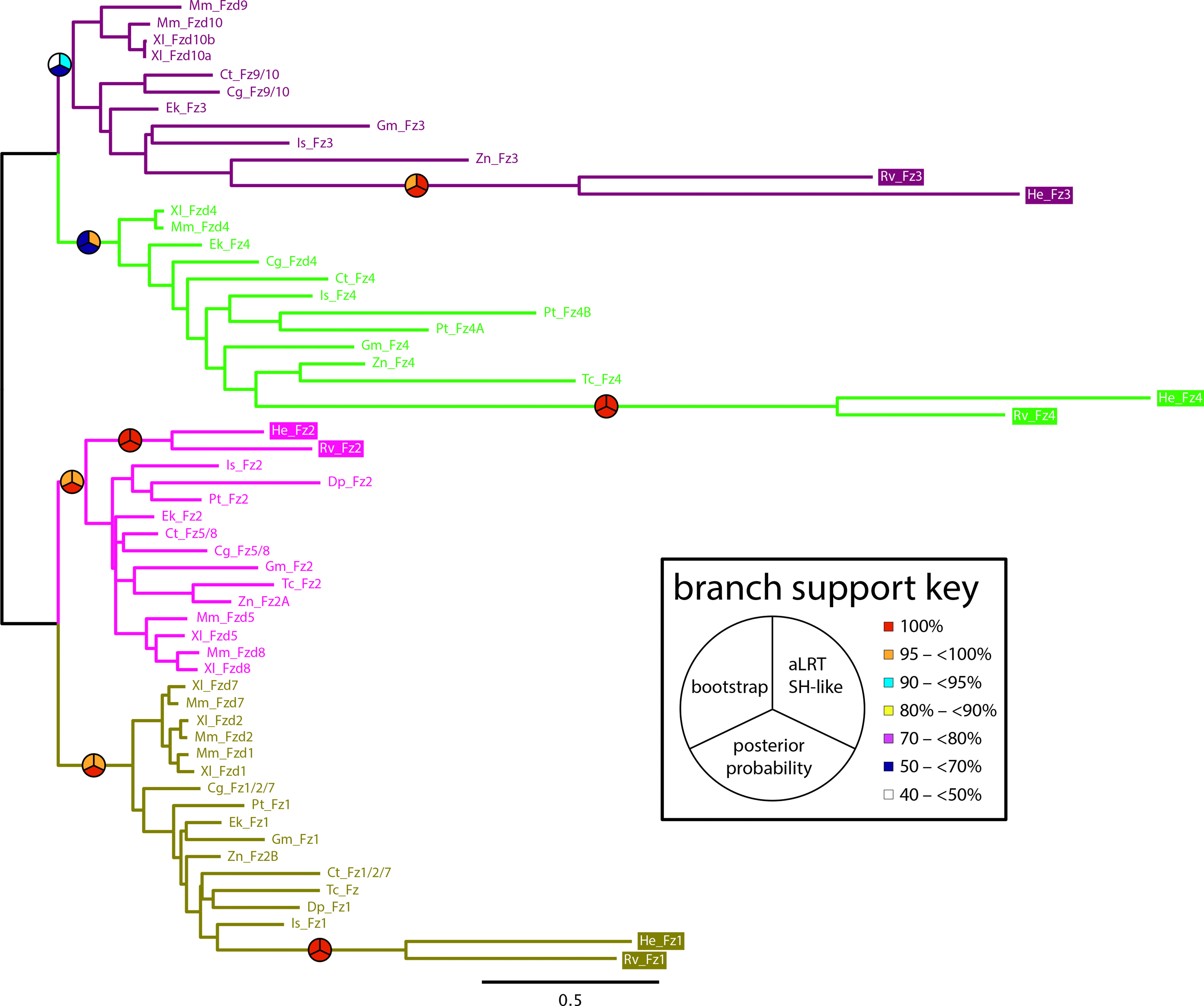
Majority rule consensus tree of Frizzled receptors. Tardigrade sequences are in colored boxes. Bootstrap supports are shown as percentages out of 500 replicates. For simplicity, only branch support values relevant to determining the identity of the candidate tardigrade Frizzled receptors are shown. Species abbreviations: Ct, *Capitella teleta*; Cg, *Crassostrea gigas*; Dp, *Daphnia pulex*; Ek, *Euperipatoides kanangrensis*; Gm, *Glomeris marginata*; He, *Hypsibius exemplaris*; Is, *Ixodes scapularis*; Mm, *Mus musculus*; Pt, *Parasteatoda tepidariorum*; Rv, *Ramazzottius varieornatus*; Tc, *Tribolium castaneum*; Xl*, Xenopus laevis*; Zn, *Zootermopsis nevadensis*

**Fig. 3:**
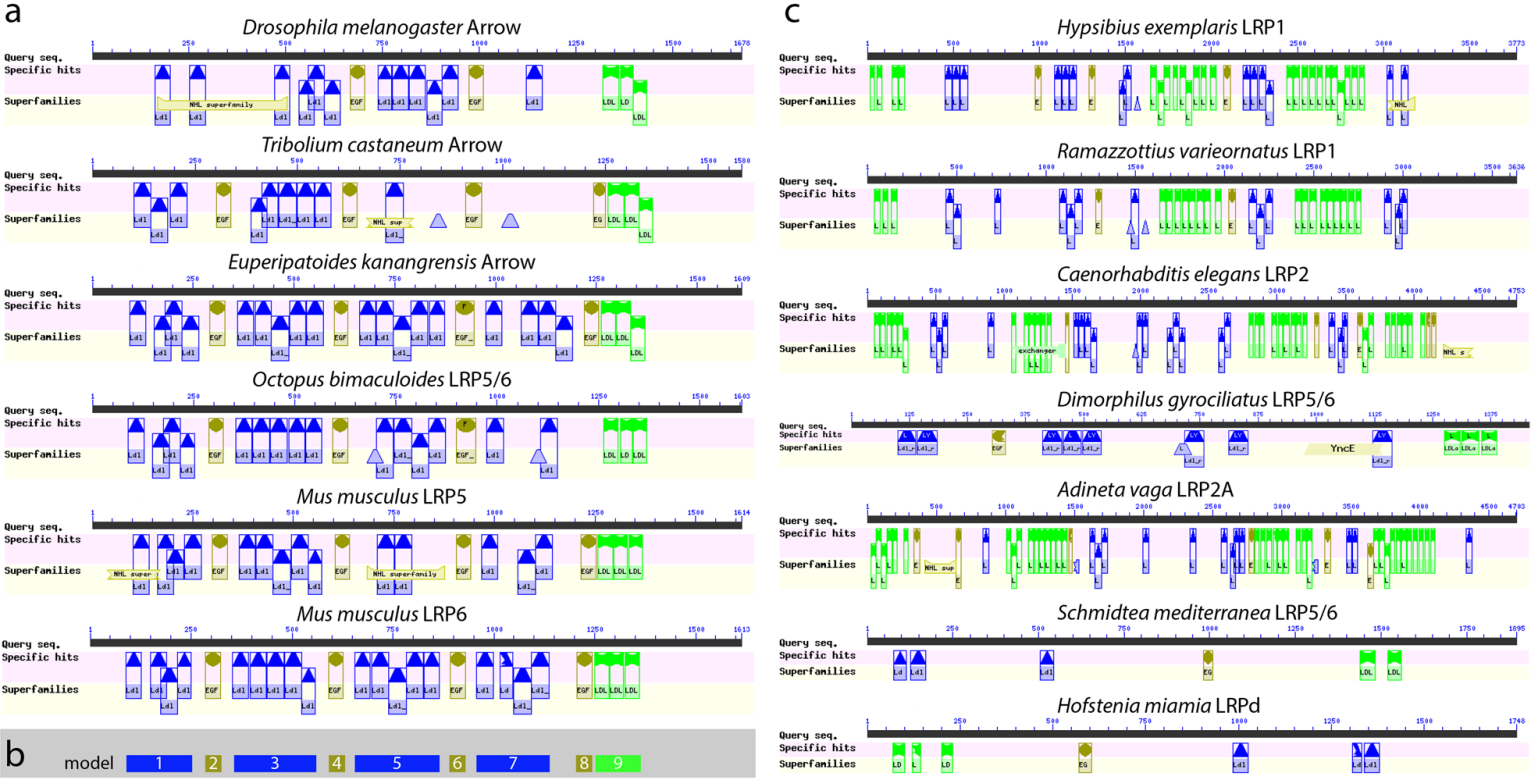
Conserved domains in LRP sequences. Blue boxes represent low-density lipoprotein receptor repeat class B domains. Gold boxes represent calcium-binding EGF-like domains. Green boxes represent low-density lipoprotein receptor class A domains. **a** Pattern of conserved domains in Arrow/LRP5/6 orthologs. **b** General pattern of conserved domains in Arrow/LRP5/6 orthologs. **c** Pattern of conserved domains in the best matches to Arrow/LRP5/6 in species that have lost several Wnt ligands. The lower case letter after the *H. miamia* sequence does not indicate specific orthology. The specific orthology of this sequence is unclear based on our analyses. The *S. mediterranea* sequence represents a highly derived Arrow/LRP5/6 ortholog based on functional analyses [98]

We were surprised that tardigrades were missing so many Wnt orthologs. This led us to wonder whether these losses were associated with modifications to the cWnt signaling pathway, a highly conserved pathway that utilizes Wnt ligands [1, 4, 11, 16]. First, we identified orthologs of *wntless*, which codes for a transmembrane transport protein that is necessary for secretion of Wnt ligands, in both species of tardigrades (Table 1). Next, we investigated the complement of Frizzled (Fz) genes, which code for receptors of Wnt ligands [86, 87]. We identified four candidate Fz genes in the genomes of both *H. exemplaris* and *R. varieornatus*. By CD-search [83], we confirmed that all candidate Fz genes encoded both a cysteine rich domain and a seven-transmembrane G protein-coupled receptor domain, domains that are indicative of Fz proteins. A phylogenetic analysis revealed that these candidates are orthologous to the four Fz genes that are found in other panarthropods (Fig. 2; Table 1). We also identified orthologs of genes that encode all major internal components of the cWnt signaling pathway [11], including *disheveled*, the β-catenin gene *armadillo* (*arm*), three β-catenin destruction complex genes—*axin*, *shaggy*, and *Adenomatous polyposis coli tumor suppressor*—and *pangolin*, which codes for a transcription factor that regulates the expression of targets of cWnt signaling (Table 1). However, we were unable to identify an ortholog of *arrow*, called *Lrp5/6* in many animals (Additional file 2: S1). This gene encodes a co-receptor of Wnt ligands, which forms a receptor complex with Fz receptors [88–90]. From N-terminus to C-terminus, *arrow* orthologs typically encode three clusters of low-density lipoprotein receptor repeat class B domains separated by calcium-binding EGF-like domains, followed by three low-density lipoprotein receptor class A domains (Fig. 3a, b). This structure was not encoded by the best tardigrade *arrow* hits (Fig. 3c).

### Analysis of Lrp sequences in other bilaterians that exhibit extensive loss of Wnt orthologs

**Fig. 4:**
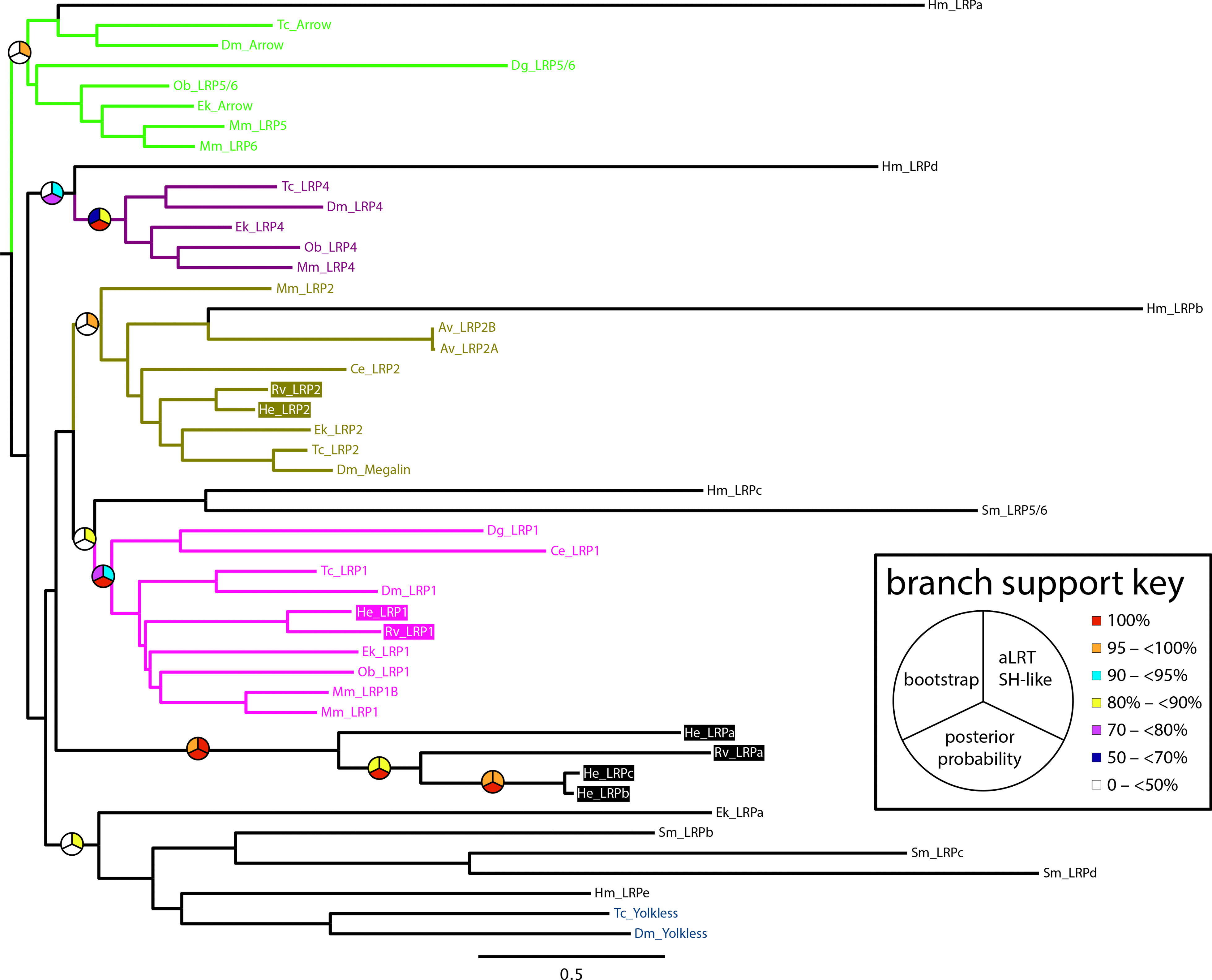
Majority rule consensus tree of LRP sequences. Tardigrade sequences are in colored boxes. Bootstrap supports are shown as percentages out of 500 bootstrap replicates. For simplicity, only branch support values relevant to determining the identity of the candidate tardigrade LRP sequences are shown. Lower case letters after taxon names indicate unclear orthology. The orthology of the *S. mediterranea* LRP5/6 sequence is based on functional analyses [98]. Species abbreviations: Ad, *Adineta vaga*; Ce, *Caenorhabditis elegans*; Dg, *Dimorphilus gyrociliatus*; Dm, *Drosophila melanogaster*; Ek, *Euperipatoides kanangrensis*; He, *Hypsibius exemplaris*; Hm, *Hofstenia miamia*; Mm, *Mus musculus*; Ob, *Octopus bimaculoides*; Rv, *Ramazzottius varieornatus*; Sm, *Schmidtea mediterranea*; Tc, *Tribolium castaneum*

To better understand the relationship between missing Wnt ligand coding genes and the absence of a clear *arrow* ortholog in tardigrades, we searched for *arrow* in the genomes of five additional non-parasitic bilaterian animals that, like tardigrades, are highly miniaturized or evolved from miniaturized ancestors [73, 74, 91, 92], and are missing several Wnt ligand-coding genes [5, 93]. We focused on free-living miniaturized animals because they may have evolved under similar selective regimes and evolutionary constraints as tardigrades. These animals included the roundworm *Caenorhabditis elegans*, the rotifer *Adineta vaga* [94], the flatworm *Schmidtea mediterranea* [95], the acoel *Hofstenia miamia* [96], and the meiobenthic annelid *Dimorphilus gyrociliatus* [97]. *D. gyrociliatus* possessed a clear ortholog of *D. melanogaster arrow* (Fig. 3c; Additional file 2: S1).* S. mediterranea* encoded a gene that was a reciprocal best BLAST hit to *D. melanogaster* Arrow (Additional file 2: S1). This gene had been identified as an ortholog of *arrow*/*Lrp5/6* in a previous study [98]. The best BLAST hits from the remaining species did not encode the pattern of protein domains that is typical of Arrow (Fig. 3c). A previous study indicated that *C. elegans* has lost an ortholog of *arrow*/*Lrp5/6*, in agreement with our results [99]. Next we performed a phylogenetic analysis of Arrow and related sequences. The *D. gyrociliatus* candidate Arrow/LRP5/6 ortholog was nested within the clade of Arrow/LRP5/6 sequences. Of the remaining miniaturized animals that we investigated, only *H. miamia* encoded a sequence that was nested within the clade of Arrow sequences, although this sequence was on a long branch (Fig. 4). Bootstrap support and posterior probability support was low for the Arrow/LRP5/6 clade, likely due to the inclusion of sequences from miniaturized animals, which were generally recovered on long branches. The best matches to *D. melanogaster* Arrow in tardigrade genomes, as determined by BLAST search (Additional file 2: S1), were recovered in a well-supported clade of LRP1 sequences (Fig. 4).

### Expression patterns of Wnt genes during tardigrade development

**Fig. 5:**
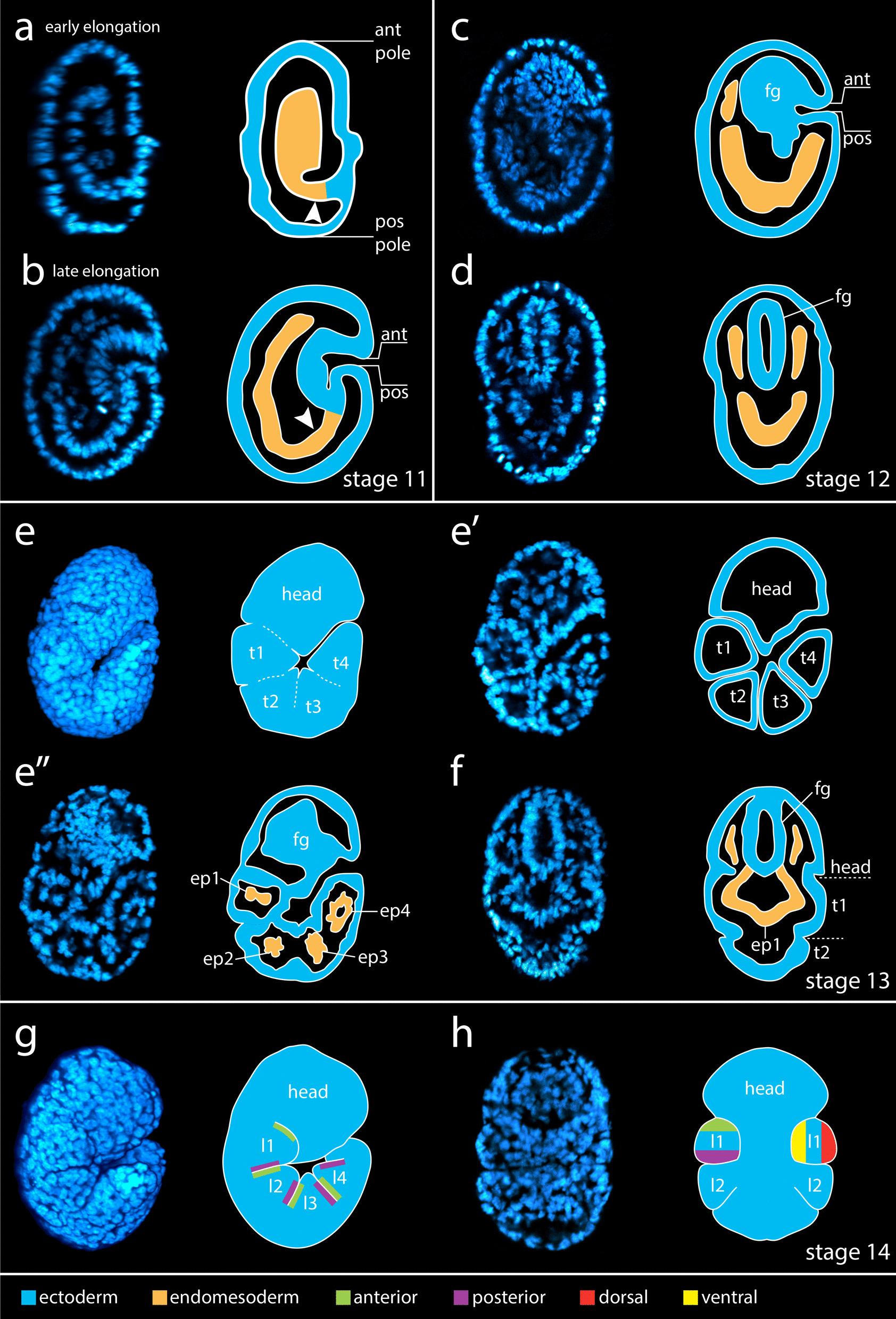
Elongation, segmentation, and leg development stages in *H. exemplaris*. Panels to the left show DAPI stained embryos. Models are provided to the right of each data panel. The key for the color-coding in models is provided at the bottom of the figure. **a** Early elongation (stage 11). **b** Late elongation (stage 11). **a**, **b** Arrowhead points to internalized cells that connect to the external ectoderm. **c**, **d** Stage 12. **e**, **f** Stage 13. **e**–**e’’** Views from more lateral to more medial. **e**, **f** Dashed lines in the model denote the position of ectodermal furrows. **g**, **h** Stage 14. Anterior is towards the top in all panels. All panels show a lateral view of embryos facing right except for **d**, **f**, and **h**, which show bilateral views. All panels show internal anatomy, except for **e** and **g**, which show external features. *ant* anterior; *ep1–ep4* endomesodermal pouch 1–endomesodermal pouch 4; *fg* foregut; *l1–l4* leg 1–leg 4; *pos* posterior, *t1–t4* trunk segment 1–trunk segment 4

**Fig. 6:**
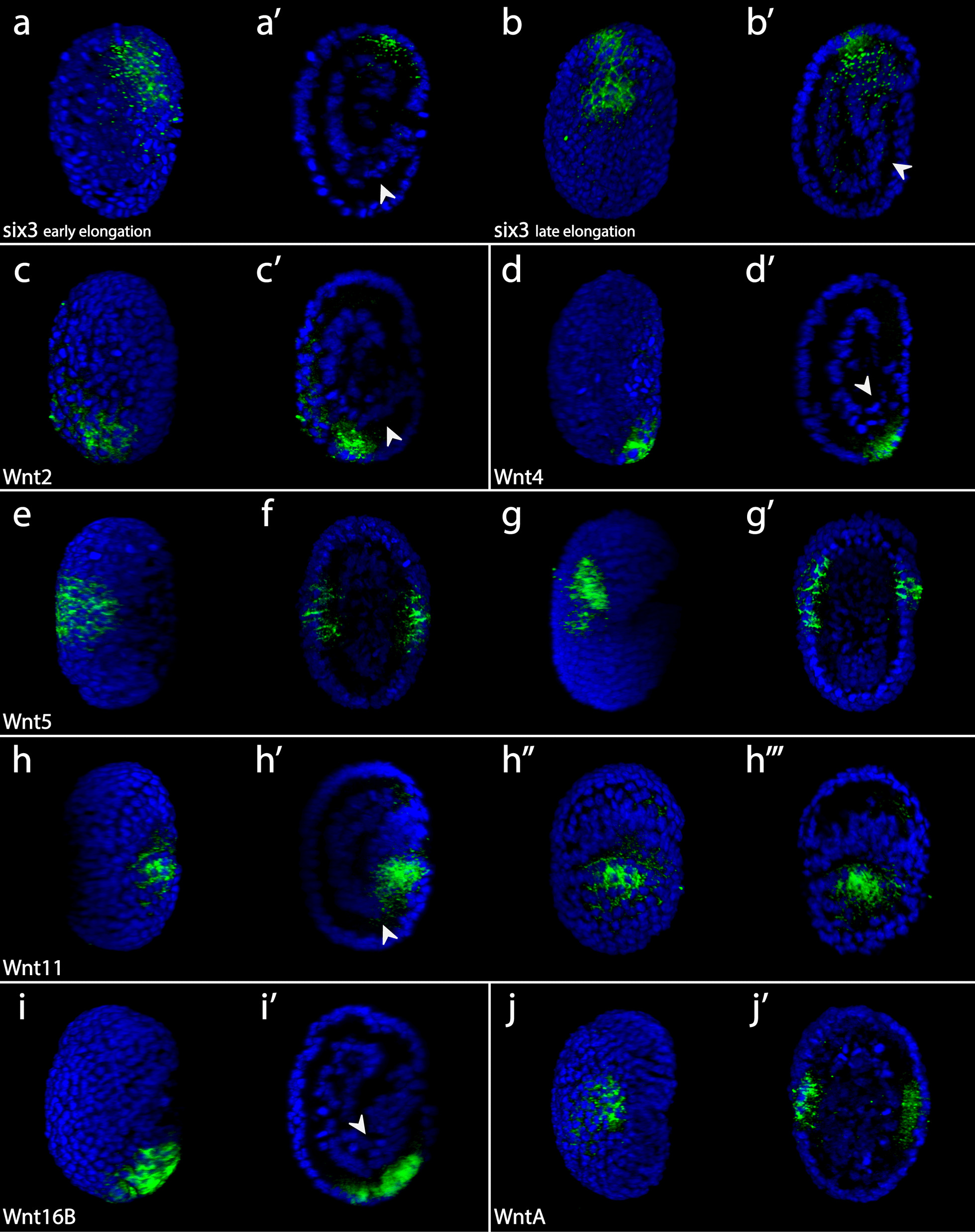
In situ hybridization results for Wnt genes in stage 11 *H. exemplaris* embryos. Green color represents gene expression. Nuclei are labeled with DAPI (blue). Images that share the same letter represent data from the same embryo. Arrowheads point to internal cells that connect to the ectoderm at the posterior end. Anterior is towards the top. **a** *Six3* expression in early elongation stage embryos. **b** *Six3* expression in late elongation stage embryos. **c** *Wnt2* expression. **d** *Wnt 4* expression. **e**, **f** *Wnt5* expression in early elongation stage embryos. **g** *Wnt5* expression in late elongation stage embryos. **h** *Wnt11* expression. **i** *Wnt16B* expression. **j** *WntA* expression. **f**, **g’**, **h’’’**, **j’** Bilateral view showing internal anatomy. **h’’** Bilateral view of outer ectoderm. All other panels show lateral views of embryos that are facing right. **a’–d’**, **h’**, **i’** show internal anatomy of laterally viewed embryos

We analyzed expression of Wnt genes at four different developmental stages in *H. exemplaris*—stage 11, the elongation stage (Fig. 5a, b), stage 12 and 13, when segmental features first appear (Fig. 5c–f), and stage 14, the leg bud stage (Fig. 5g, h) [100]. *He-Wnt9* was broadly expressed across embryos at all embryonic stages that we investigated, but may exhibit strongest expression in the endomesodermal layer (Additional file 3: Fig. S2e–h). We did not detect clear *He-Wnt16A* expression at any stage that we investigated (Additional file 3: Fig. S2m–p). The AP axis forms by a process referred to as elongation during stage 11 in *H. exemplaris—*a process during which the entire AP axis forms simultaneously, rather than forming in anteroposterior order as would be indicative of posterior growth (Fig. 5a, b) [100]. We analyzed *He-six3* expression, a marker of anterior identity [76, 101], with in situ hybridization at stage 11 to distinguish between the anterior and posterior embryonic poles (Fig. 6a, b). In stage 11 embryos, an internalized cell layer extended to the external ectodermal cell layer at one embryonic pole (Fig. 5a, b). *Six3* was expressed at the opposite pole (Fig. 6a’, b’), indicating that the internalized cells connect to the ectoderm at the posterior side of stage 11 embryos. At stage 11, *He-Wnt2*, *He-Wnt4*, and *He-Wnt16B* were expressed in the ectodermal layer near the posterior embryonic pole (Fig. 6c, c’, d, d’, i, i’). *He-Wnt11* appeared broadly expressed at this stage, but the strongest signal was located near the presumptive posterior end of the embryo (Fig. 6h–h’’’; Additional file 3: Fig. S2i). The expression domains of *He-Wnt5* and *He-WntA* were localized to the ectodermal layer, between the anterior and posterior poles of embryos (Fig. 6e–g’, j, j’). *He-Wnt5* expression extended across the presumptive dorsoventral axis, whereas *He-WntA* expression was restricted to the presumptive ventrolateral region of embryos. The *He-Wnt5* expression domain shifted to a slightly more anterior location in late stage 11 embryos (Fig. 6g, g’), which may be associated with movement of cells during the elongation process.

**Fig. 7:**
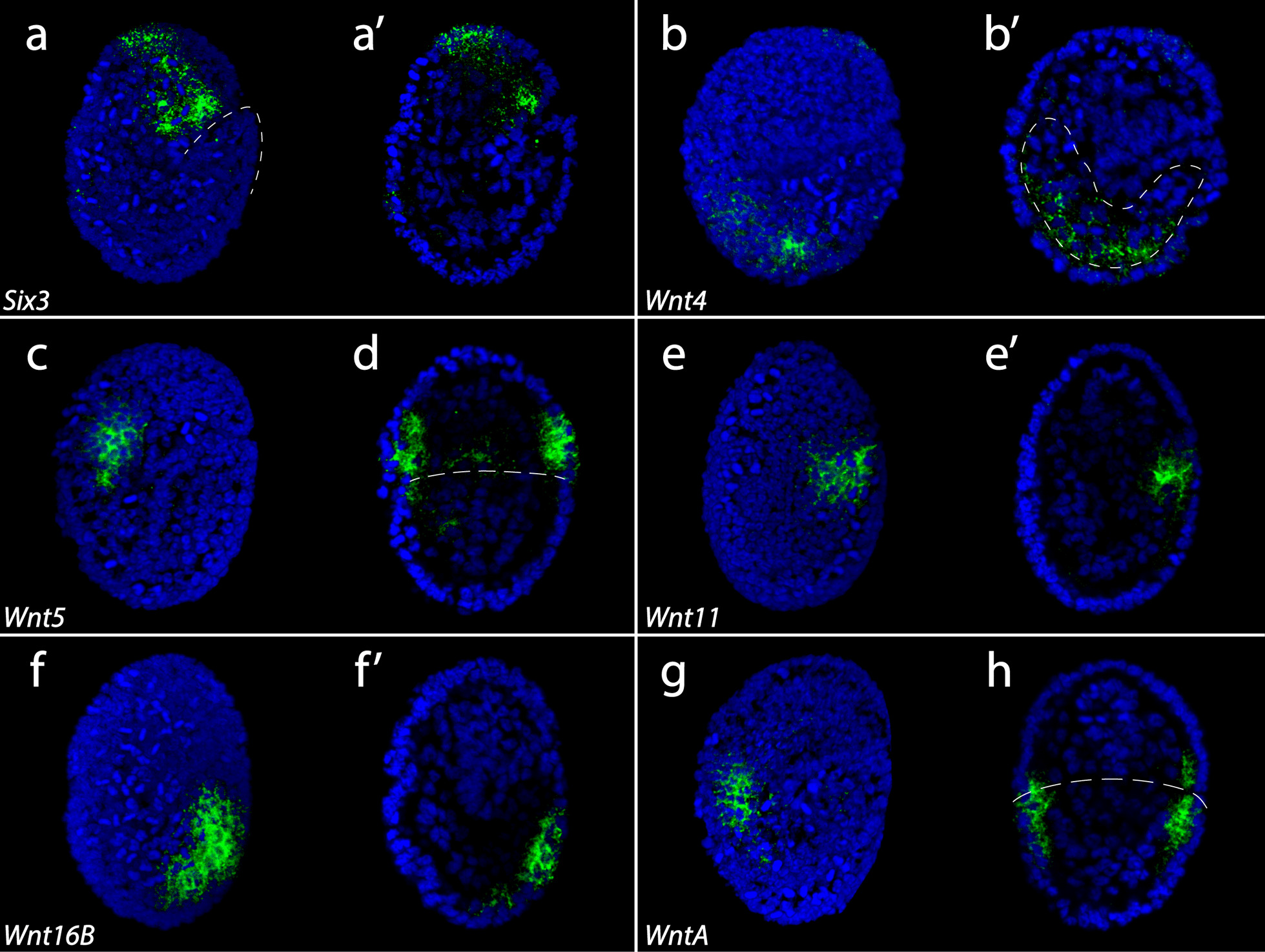
In situ hybridization results for Wnt genes in stage 12 *H. exemplaris* embryos. Green color represents gene expression. Nuclei are labeled with DAPI (blue). Images that share the same letter represent data from the same embryo. Anterior is towards the top. **a** *Six3* expression. Dashed line outlines the posterior tip. **b** *Wnt4* expression. **c**, **d** *Wnt5* expression. **e** *Wnt11* expression. **f** *Wnt16B* expression. **g**, **h** *WntA* expression. All panels show lateral views of embryos that are facing right except for **d** and **h** which are bilateral views showing internal anatomy. **a’**, **b’**, **e’**, **f’** show internal anatomy of laterally viewed embryos. **b’** Dashed lines outline developing endomesodermal pouches. **d**, **h** Dashed line demarcates the boundary between the head and the trunk

After elongation, the AP axis is curved in a characteristic “C” shape. At stage 12, the developing foregut in the head and trunk endomesoderm is visible (Fig. 5c, d) [100]. We did not detect strong signal for *He-Wnt2* at this stage or later stages (Additional file 3: Fig. S2a–c).* He-Wnt4* was expressed in the endomesodermal layer of the developing trunk (Fig. 7b, b’). *He-Wnt5* was expressed in the ventrolateral ectoderm of the posterior head (Fig. 7c, d). *He-Wnt11* signal was detected broadly across embryos at stage 12, but the strongest expression was localized to the posteriormost region of developing embryos (Fig. 7e, e’; Additional file 3: Fig. S2j). *He-Wnt16B* was expressed broadly across the posterior half of the developing trunk, excluding the posteriormost region (Fig. 7f, f’). *He-WntA* was expressed in the ectodermal layer in the anteriormost region of the developing trunk and posteriormost part of the head (Fig. 7g–h).

**Fig. 8:**
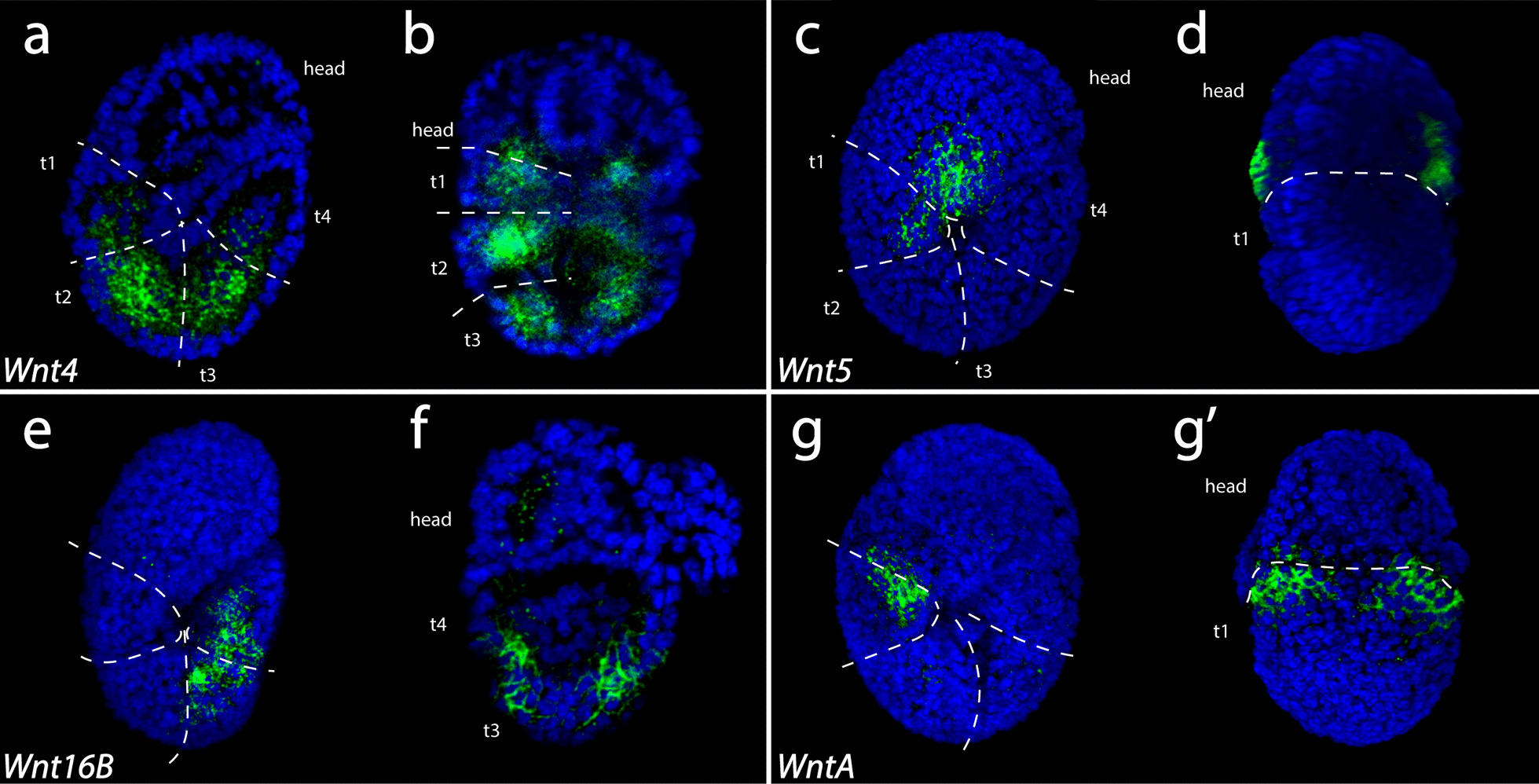
In situ hybridization results for Wnt genes in stage 13 *H. exemplaris* embryos. Green color represents gene expression. Nuclei are labeled with DAPI (blue). Images that share the same letter represent data from the same embryo. Anterior is towards the top. **a**, **b** *Wnt4* expression. **c**, **d** *Wnt5* expression. **e**, **f** *Wnt16B* expression. **g**, **g’** *WntA* expression. **a**, **c**, **e**, **g** Lateral view of embryos that are facing right. **d**, **g’** Bilateral views showing external anatomy.** f **Bilateral view showing internal anatomy. Dashed line demarcates the boundary between the head and the trunk. **a**, **b**, **c**, **e**, **g** Dashed lines demarcate segment boundaries. *t1–t4* trunk segment 1–trunk segment 4

At stage 13, segmentation is clearly visible in the form of ectodermal furrows and endomesodermal pouches (Fig. 5e, f) [100]. *He-Wnt4* was expressed strongest in the endomesodermal layer of the developing trunk (Fig. 8a, b). At stage 13, *He-Wnt5 *expression looked very similar to its expression at stage 12. At this stage, *He-Wnt5* was primarily expressed in a ventrolateral region of the ectodermal layer of the developing head (Fig. 8c, d). *He-Wnt11* was expressed broadly across developing embryos (Additional file 3: Fig. S2k). *He-Wnt16B* was expressed in the lateral ectoderm of the third and fourth trunk segment, excluding the posteriormost region of the fourth trunk segment (Fig. 8e, f). *He-WntA* was expressed in a dorsolateral stripe in the boundary between the head and the first trunk segment (Fig. 8g, g’).

**Fig. 9:**
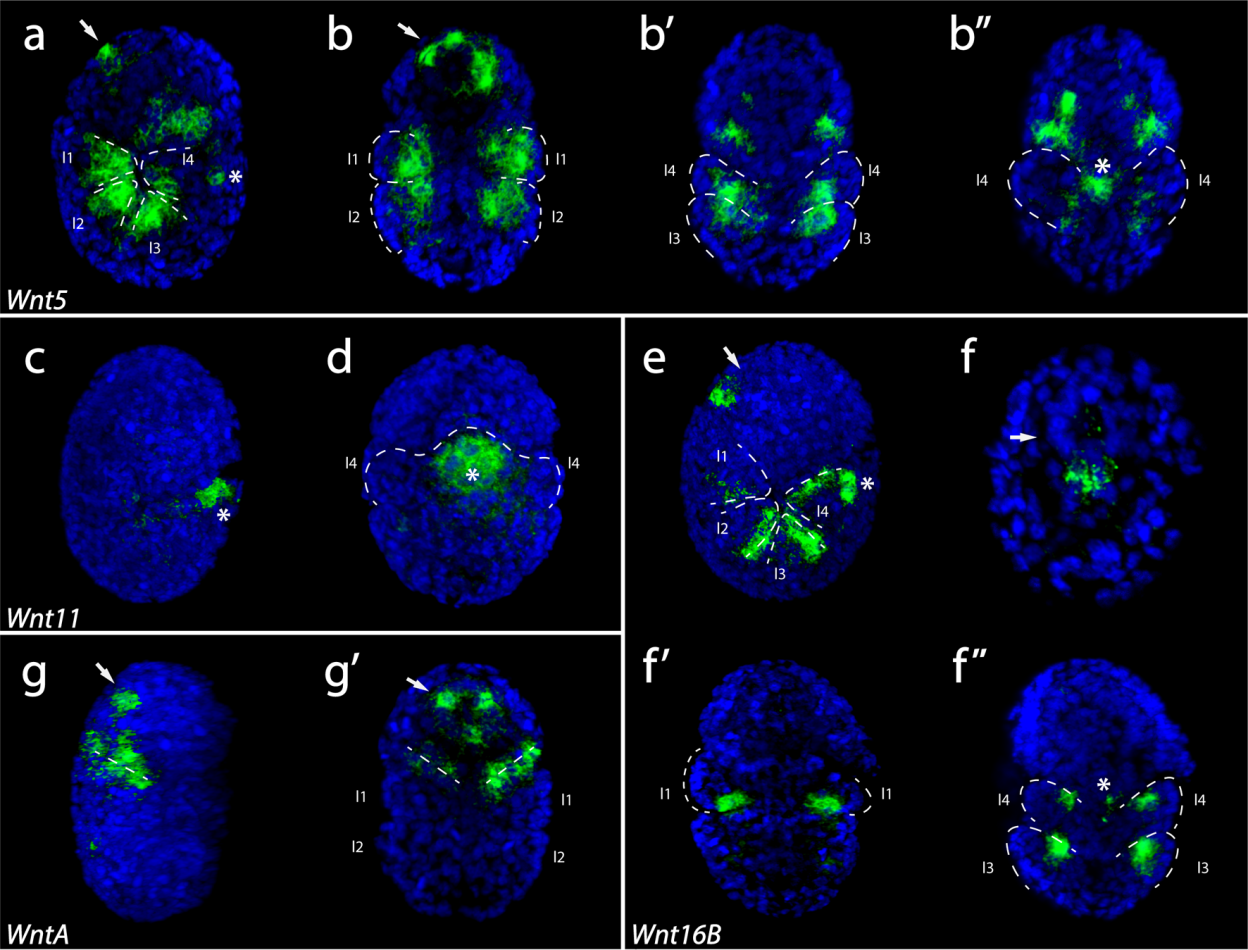
In situ hybridization results for Wnt genes in stage 14 *H. exemplaris* embryos. Green color represents gene expression. Nuclei are labeled with DAPI (blue). Images that share the same letter represent data from the same embryo. Arrows point to the developing pharynx. Asterisks mark the region between the posteriormost legs. Anterior is towards the top. **a**, **b** *Wnt5* expression. **a** Ventral surface of legs is in view. **b**, **b’** Dorsal surface of legs is toward the outside and the ventral surface is toward the inside of the embryo. **c**, **d** *Wnt11* expression. **d** Bilateral view. The posterior end of embryo is outlined. **e**, **f** *Wnt16B* expression. **g** *WntA* expression. **a–b’’**, **e**, **f’**, **f’’** Dashed lines outline legs. **a**, **c**, **e**, **g** Lateral view of embryos that are facing right. **b–b’’**, **f–f’’**, **g’** Bilateral view showing internal anatomy. **g**, **g’** Dashed lines demarcate the boundary between the head and the trunk. *l1–l4* leg 1–leg 4

At stage 14, developing legs are clearly visible (Fig. 5g, h). Other features, such as pharynx and trunk ganglia are also visible [100]. At this stage, *He-Wnt4* was very weak or absent (Additional file 3: Fig. S2d). *He-Wnt5* was expressed broadly across the inner surface of developing legs, between the developing hind legs, in the ventrolateral ectoderm of the head, and in the developing pharynx (Fig. 9a–b’’). *He-Wnt11* exhibited strongest expression in the posteriormost region of the AP axis, between the developing hind legs, at this stage (Fig. 9c, d). We also detected staining for *He-Wnt11* in other parts of the embryo (Additional file 3: Fig. S2l). At stage 14, *He-WntA* was expressed in a dorsolateral ectodermal stripe between the head and first trunk segment, and in the developing pharynx (Fig. 9g, g’). *He-Wnt16B* was expressed in a stripe in each developing leg (Fig. 9e, f’, f’’). We also detected expression of this gene in the posteriormost region between the developing hind legs (Fig. 9e, f’’), and near the posterior part of the pharynx, which likely represents the esophagus (Fig. 9f).

## Discussion

### Evolutionary dynamics of Wnt genes in Tardigrada

**Fig. 10:**
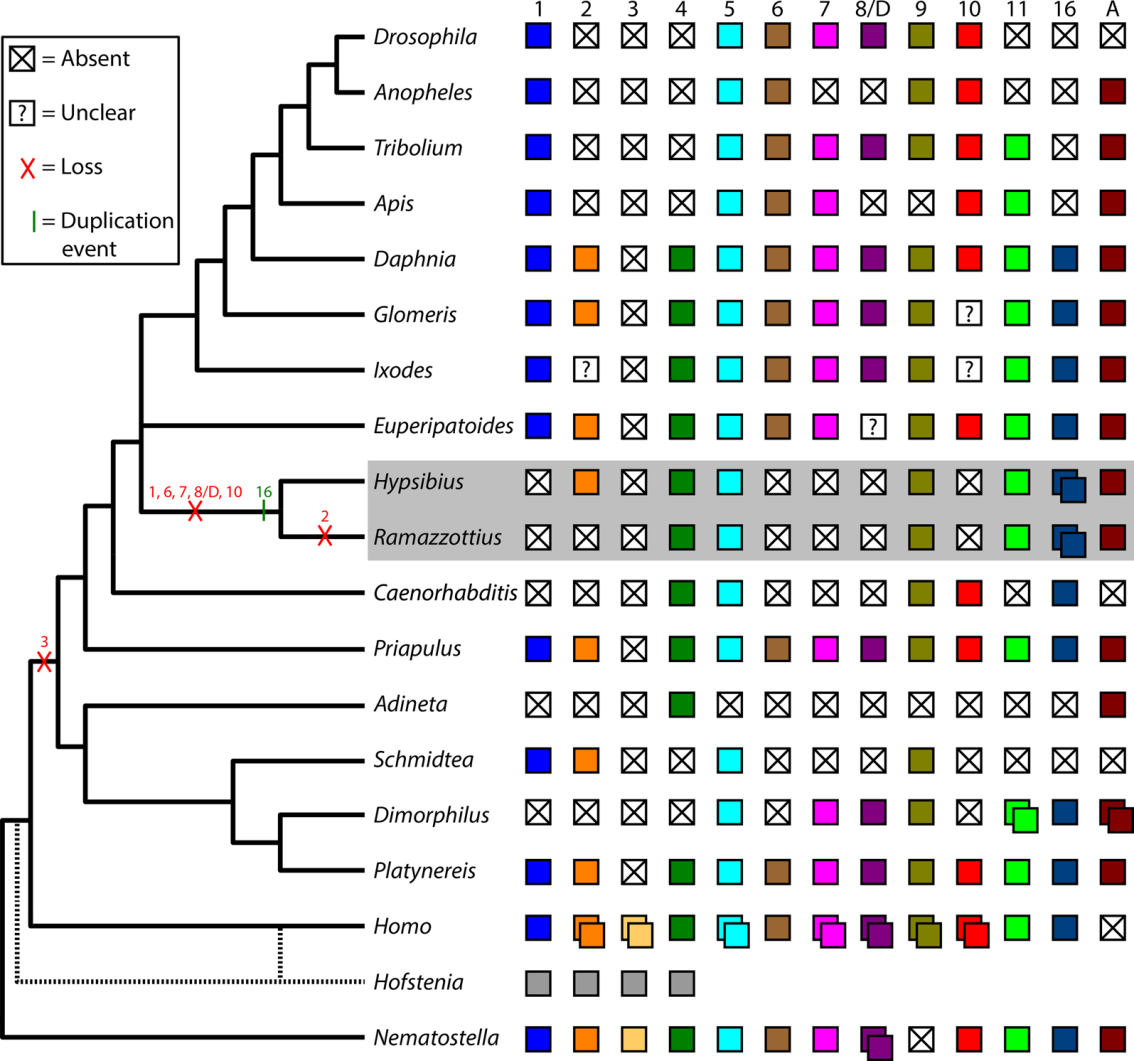
Summary of distribution of Wnt orthologs in metazoan genomes. “X” in white boxes indicates the loss of a Wnt gene. “?” in white boxes indicates that a fully sequenced genome is unavailable for the associated lineage, so it is unclear whether the ortholog has been lost, or is present, but unsequenced. Gray boxes associated with *Hofstenia* indicate unclear orthology of the four Wnt genes found in the genomes of representatives of this lineage. Dashed lines coming off of *Hofstenia* represent different hypotheses of the relationship of this lineage with other bilaterians. The interrelationships of panarthropod phyla (Arthropoda, Onychophora, Tardigrada) are depicted as a polytomy because they are not currently resolved [69]. Arthropoda = *Drosophila*–*Ixodes*; Onychophora = *Euperipatoides*; *Hypsibius–Ramazzottius* = Tardigrada

Orthologs of *Wnt2*, *Wnt4*, *Wnt5*, *Wnt9*, *Wnt11*, *Wnt16*, and *WntA* are conserved in Tardigrada (Fig. 1). By contrast, *Wnt1*, *Wnt6*, *Wnt7*, *Wnt8*, and *Wnt10* are missing in the genomes of two tardigrade species, *H. exemplaris* and *R. varieornatus*. These genes were most likely lost specifically within the tardigrade lineage (Fig. 10). However, these two tardigrade species are fairly closely related [78]. Therefore, whether these genes were lost in an ancestor of all tardigrades, or within crown group Tardigrada is unclear. Likewise, it is difficult to place the duplication event that gave rise to *Wnt16* paralogs. It is clear that this duplication event occurred somewhere in the tardigrade lineage, because tardigrade *Wnt16* genes formed a well-supported monophyletic group in our phylogenetic analyses (Fig. 1). It is also clear that these genes evolved by a duplication event that occurred in an ancestor of both *H. exemplaris* and *R. varieornatus*, rather than by independent duplication events (Fig. 10). The fact that the closest relative of *He-Wnt16A* is *Rv-Wnt16A* and the closest relative of *He-Wnt16B* is *Rv-Wnt16B* supports this conclusion (Fig. 1). As with the losses of Wnt genes, this duplication event could have occurred in any common ancestor of *H. exemplaris* and *R. varieornatus* in the tardigrade lineage. Genomic data from more distantly related tardigrade species would enable a more precise phylogenetic resolution of tardigrade specific losses and duplication of Wnt genes. By contrast, we can more precisely resolve the absence of *Wnt2* in the genome of *R. varieornatus* as a loss in the lineage leading to this species after it split from the lineage leading to *H. exemplaris*, because *Wnt2* is retained in *H. exemplaris* and outgroups of Tardigrada (Fig. 10).

Additionally, our results suggest that Wnt genes are dispersed throughout a chromosome or several chromosomes in the tardigrade species that we investigated, rather than clustered like Wnt genes in some other animal genomes [2, 5, 59]. The dispersion of Wnt genes in the genome most likely represents a derived state and could be related to the extensive loss of Wnt genes. Dispersion of ancestrally clustered paralogs in tardigrade genomes has been previously suggested for Hox genes and NK homeobox genes [75, 102]. Therefore, dispersion of ancestrally clustered paralogs of developmental genes could represent a general pattern of genome evolution in the tardigrade lineage. The evolution of rapid embryogenesis has been suggested to abrogate purifying selection that would otherwise maintain clustered Hox genes and Parahox genes [103], and may also explain the dispersion of Wnt genes and NK homeobox genes in tardigrade genomes. Additionally, dispersion of ancestrally clustered paralogous developmental genes has been suggested to represent a general outcome of miniaturization [74].

In contrast to the extensive loss of Wnt orthologs, we identified nearly all other components of the cWnt signaling pathway in tardigrades (Table 1). This was not surprising given that cWnt signaling regulates many developmental processes in other animals. However, we were unable to identify an ortholog of *arrow* (Additional file 2: S1), referred to as *Lrp5/6* in many animals, which codes for a co-receptor of Wnt ligands [88–90, 104]. The closest match to *arrow* in tardigrades is a gene that most likely codes for an *Lrp1* ortholog (Figs. 3c, 4). Possibly, the Wnt ligands that were lost in Tardigrada represent the Wnt ligands that required Arrow as a co-receptor to activate cWnt signaling in the ancient ancestors of tardigrades. To test this hypothesis, more studies are required to determine exactly which Wnt ligands require Arrow for cWnt signaling broadly across Metazoa.

### Potential correlates of Wnt gene loss in Tardigrada

Barring initial redundancy, which is a poor explanation for long-term retention of Wnt paralogs [3], the loss of Wnt genes in the tardigrade lineage is most likely associated with modifications to development in Tardigrada. However, identifying exactly how development has been modified in tardigrades is extremely difficult, especially given the relatively scarce functional data for most Wnt genes. Additionally, we should not draw strong conclusions based on the loss of several Wnt genes in Tardigrada, given that gene loss is very common in this lineage [97, 105], and given that the loss of one or more Wnt gene(s) is fairly common across Metazoa [3]. Nonetheless, a comparative approach may help explain the extensive loss of Wnt genes in Tardigrada. First, we note that, in addition to tardigrades, several other secondarily miniaturized animals exhibit extensive loss of Wnt genes (Fig. 10, *Caenorhabditis*, *Adineta*, *Schmidtea*, *Dimorphilus*, *Hofstenia*) [3, 5, 97, 106, 107]. The extensive loss of Wnt genes in some secondarily miniaturized animals may be due to the evolution of simpler cell fate specification mechanisms related to reduction in cell number and simplified morphology that often accompanies miniaturization. Along these lines, our expression data suggest that combinatorial interactions among Wnt genes may play a much less important role during development of tardigrades compared to typical macroscopic animals. In arthropods, onychophorans, and annelids, several Wnt genes are coexpressed in the posterior growth zone and are expressed in similarly positioned segmentally reiterated stripes [3, 28, 108]. Although Wnt genes exhibit different expression patterns during appendage development between arthropods and onychophorans (see below), within each lineage, several Wnt genes exhibit very similar expression patterns during appendage development [3, 28, 63]. Similar expression patterns of different Wnt genes in arthropods, onychophorans, and annelids most likely reflect combinatorial interactions of these genes during development [3]. Necessary combinatorial interactions of several Wnt paralogs could partly explain their conservation across much of Metazoa. None of the Wnt genes in *H. exemplaris* exhibited highly similar expression patterns during segmentation or leg development. Therefore, combinatorial interactions between Wnt orthologs must be less important during development of tardigrades. In some secondarily miniaturized animals, like tardigrades, one or more Wnt genes may become superfluous or redundant with a reduced requirement of combinatorial interactions, ultimately leading to loss by neutral evolutionary processes.

A second non-mutually exclusive possibility is that the loss of one or more Wnt genes in Tardigrada is related to the loss of posterior growth, a process that is regulated by cWnt signaling in many macroscopic animals [20, 21, 27, 109, 110]. As with tardigrades, posterior growth is reduced, absent, or highly modified in many secondarily miniaturized animals ([79, 100, 111, 112], Martín-Durán JM, pers. comm.), which have also lost several Wnt genes (Fig. 10, *Caenorhabditis*, *Adineta*, *Schmidtea*, *Dimorphilus*, *Hofstenia*). Additionally, long germband insects may retain fewer ancestral Wnt genes than their short germband relatives that continue to utilize posterior growth (Fig. 10, compare the long germband insects *Drosophila*, *Anopholes*, and *Apis* to the shortband insect *Tribolilum*) [3]. *Arrow*/*Lrp5/6* is also necessary for normal posterior growth in arthropods and vertebrates [54, 113–115]. Therefore, the loss of *arrow*/*Lrp5*/*6* in tardigrades and several other miniaturized animals may represent an additional genomic signature of the loss of posterior growth in these animals.

Although feasible, there are significant difficulties associating the loss of Wnt genes and *arrow*/*Lrp5/6* with the loss of posterior growth. First, the loss of *arrow*/*Lrp5/6* is not tightly associated with the loss of Wnts genes or posterior growth. For example, *D. gyrociliatus* retains an *arrow*/*Lrp5/6* ortholog (Figs. 3c, 4; Additional file 2: S1), but is missing several Wnt genes (Fig. 10, *Dimorphilus*) [97] and lacks posterior growth (Martín-Durán JM, pers. comm.). Likewise, although long germband insects have lost several Wnt genes and do not utilize posterior growth, they retain *arrow* [88]. Therefore, *arrow* clearly plays important developmental roles besides regulating posterior growth. Evidence for an additional role of *arrow*/*Lrp5/6* comes from studies of the flatworm *S. mediterranea*. In this species, this gene is required to regulate posterior cell fate and proliferation during AP axis regeneration [98]. Second, given the sparse data available in regards to which Wnt gene regulates posterior growth, and the apparent interchangeability of Wnt gene function in this process based on data that are available [26, 58], it is not possible to associate with confidence the loss of any particular Wnt gene in tardigrades with the loss of posterior growth. Nonetheless, based on a comparative perspective, it remains possible that reduced combinatorial interactions and the loss of posterior growth, both associated with miniaturization, contributed to the loss of several Wnt genes and *arrow*/*Lrp5/6* in Tardigrada.

### Wnt genes and regionalized AP cell fate specification

Several Wnt genes appeared to be expressed in regionalized patterns along the developing AP axis during embryogenesis in *H. exemplaris*. Given the absence of segment markers during elongation, it is difficult to determine precisely where the Wnt genes are expressed during this stage relative to each other (Fig. 6). However, the relative order of expression along the AP axis of some Wnt genes appeared to be maintained between the elongation stage and later stages during which segmental features are developing (Figs. 7, 8, 9). In other bilaterians, Wnt genes play a critical role in establishing the AP axis by promoting posterior identity, in part, by repressing anterior identity [9, 15–19]. We did not detect expression of any Wnt genes at the anterior pole of embryos at the elongation stage, besides *He-Wnt9* and *He-Wnt11*, genes that exhibited very broad expression patterns. Therefore it is possible that some Wnt genes are playing roles in promoting posterior identity in *H. exemplaris* and other tardigrades. Interestingly, several Wnt genes are expressed in regionalized AP patterns in the onychophoran *Euperipatoides kanangrensis* during early stages of segmentation [28]. Based on these regionalized patterns, it has been suggested that Wnt genes may specify segment identities in onychophorans [28]. Potentially, one or more Wnt genes are playing a role in specifying segment identities in tardigrades. Future functional studies may help resolve this issue.

### Wnt genes and segment polarity

In many arthropods, *wg*, *en*, and *hh*, interact positively as part of a regulatory network to establish segment polarity [30–34, 43–45, 50, 52, 57]. A segmentally reiterated pattern of gene expression emerges, which includes a stripe of *wg* expression positioned immediately in front of a stripe of *en* and *hh* expression. Parasegmental boundaries develop between the stripes of *wg* and the stripes of *en* and *hh* expression. Parasegmental boundaries are then replaced by segmental boundaries, which develop posterior to the *en* and *hh* stripes. In spiders, a different Wnt gene most likely substitutes for *wg* to regulate segment polarity [58]. Segment polarity genes have been investigated in two onychophoran species, *Euperapatoides kanangrensis* [64, 65] and *E. rowelli* [66]. The expression patterns of the segment polarity genes in onychophorans are remarkably similar to those in arthropods. However, in contrast to arthropods, in onychophorans, Wnt genes and other segment polarity genes, besides *en* in *E. kanangrensis*, are first expressed in stripes after the earliest signs of segmentation appear [64, 66]. Therefore, unlike in arthropods, *wg* and other Wnt genes most likely do not play a role in segment formation in onychophorans [28, 64, 66]. Nonetheless, a conserved segment polarity network is most likely regulating intrasegmental patterning in onychophorans after segments develop [65, 66].

In *H. exemplaris*, the first signs of segmentation are the formation of endomesodermal pouches [100]. *He-*En expression is first detected in the ectoderm at a later stage in segmentally reiterated stripes immediately anterior to where ectodermal furrows develop between the underlying endomesodermal pouches [116]. Our results suggest that *wg* has been lost in the tardigrade lineage. Therefore, if cWnt signaling regulates *en* expression to establish segment polarity in the tardigrades of our study, this interaction must be mediated though a different Wnt gene. No Wnt gene was expressed in stripes in the ectoderm at the earliest stages of the segmentation process in *H. exemplaris* (Figs. 7, 8). It is possible that cWnt signaling is not required for maintaining *en* expression during segment formation in Tardigrada. However, cWnt signaling could be maintaining *en* expression during segment formation in Tardigrada via a slightly modified mechanism. *He-Wnt4* was expressed broadly throughout the endomesodermal layer of the trunk during segment formation (Figs. 7b’, 8a, b), rather than in the ectoderm as would be expected for a Wnt gene interacting with *en*. Nonetheless, the ligand that *He-Wnt4* encodes could be providing a signal to En expressing cells. No other Wnt gene was expressed in a segmentally reiterated pattern that would be indicative of a role in segment formation. The only Wnt gene that was expressed in a stripe-like pattern in *H. exemplaris* was *He-Wnt16B*, which was expressed later in developing legs (Fig. 9e, f’, f’’). Although this gene is unlikely to be playing a role in segment formation, its stripe-like expression pattern may indicate a later acting segment polarity function. However, by the time that legs are visible, *He-*En is no longer expressed in segmentally reiterated stripes [116]. Taken together, our results raise the interesting possibility that Wnt genes may not play a role in regulating segment polarity via regulatory interactions with *en* in Tardigrada. The precise developmental roles of *Wnt4*, *Wnt16B*, and other Wnt genes need to be clarified before a strong conclusion can be drawn regarding a role of cWnt signaling in regulating segment polarity, or the lack thereof, in Tardigrada. Additionally, other commonly conserved components of the segment polarity network, such as *hh*, need to be investigated in tardigrades. Resolving these issues is critical for determining the antiquity of the roles that the segment polarity network plays during development in Arthropoda and Onychophora.

### Wnt genes and leg development

In arthropods, Wnt genes also play important roles in regulating appendage development. First, *wg* activates expression of *Distal-less* (*Dll*) to initiate appendage outgrowth [46, 51, 117, 118]. *Dll* most likely also regulates appendage growth in onychophorans [119, 120]. *Wg* and several other Wnt genes are expressed in a distal pattern in developing appendages in *E. kanangrensis*, suggesting that *wg*, and potentially other Wnt genes, may also be regulating growth via *Dll* in onychophorans [28, 120]. *Dll* is expressed broadly across the developing legs of *H. exemplaris*, and most likely plays a role in regulating leg outgrowth in Tardigrada [82]. The tardigrades we analyzed appear to have lost *wg*. Therefore, *wg* cannot be activating *Dll* expression in these species. It is possible that Wnt signaling is not required for activating *Dll* expression in Tardigrada. Alternatively, one or more other Wnt genes may play this role. *Wnt4* is a potential candidate for regulating *Dll* expression in tardigrades. In *H. exemplaris*, this gene is expressed in the endomesodermal layer below where legs will develop in the overlying ectoderm (Fig. 8a, b). Two additional strong candidates are *Wnt5* and *Wnt16B* (discussed in more detail below), which are both expressed strongly in developing legs (Fig. 9a–b’, e, f’, f’’). Functional studies are required to determine whether these Wnt genes or others are required to activate *Dll* expression in Tardigrada.

Later in appendage development in arthropods, *wg* specifies ventral appendage fate [40, 42, 46, 51, 121–123]. As with *wg*, several other Wnt genes are expressed in the developing ventral leg domain in arthropods and likely play a redundant or combinatorial role in specifying ventral appendage fate [3, 60, 62, 63]. Unlike in arthropods, no Wnt genes are expressed in the ventral appendage domain in onychophorans [28]. It has been suggested that ventral expression of Wnt genes in developing appendages evolved in the arthropod lineage [28]. The tardigrades that we studied lack a *wg* ortholog, so this gene cannot be playing a role in establishing ventral appendage fate in these species. However, in *H. exemplaris*, *He-Wnt5* is expressed on the inner side of developing legs (Fig. 9a–b’). Although not definitive, we interpret the inner side of the developing legs as ventral (Fig. 5h). In this interpretation, *He-Wnt5* may specify ventral appendage fate in *H. exemplaris*. *Wnt5* is not expressed in a ventral stripe in developing legs of arthropods [3, 58, 63]. This may indicate that a role of *Wnt5* in specifying ventral fates in developing *H. exemplaris* legs, even if present, is not homologous to the function that Wnt genes play in specifying ventral fates in arthropod appendages.

We detected expression of *He-Wnt16B* in a stripe in each developing leg (Fig. 9e, f’, f’’). Although not definitive, these stripes may lay in the posterior region of each developing leg (Fig. 5g, h). The insects that have been studied most extensively, *D. melanogaster* and *T. castaneum*, lack a *Wnt16* ortholog [3]. In chelicerates and the millipede *Glomeris marginata*, *Wnt16* is expressed in the distal tip and a ventral stripe, or just in a ventral stripe, in developing legs [3, 58, 63]. The *Wnt16* ortholog of the onychophoran *E. kangrenesis* is expressed at the distal tip and in a posterior stripe in developing legs and other appendages, besides the frontal appendage, in which it is only expressed in a posterior stripe [28]. It is possible that a posterior stripe of *Wnt16* in developing appendages was inherited in tardigrades and onychophorans from the last common ancestor of these lineages. Depending on the interrelationships of the panarthropod phyla (reviewed in [69]), this ancestor could represent the last common ancestor of Panarthropoda, in which case, the absence of a posterior stripe of *Wnt16* in developing arthropod appendages would represent a derived state of Arthropoda.

## Conclusions

Studies of tardigrades hold the potential to help resolve the evolution of developmental mechanisms in Panarthropoda. Our study revealed interesting possibilities regarding the evolution of the roles of Wnt signaling in regulating the development of key features of Panarthropoda—segmentation and appendages. Although in many respects the anatomy of tardigrades may best represent the anatomy of the last common ancestor of Panarthropoda [124, 125], we cannot assume that developmental mechanisms in Tardigrada represent ancestral panarthropod mechanisms. In fact, studies indicate that tardigrade development is highly derived with simplification representing a common theme of developmental evolution in this lineage. For example, tardigrades have lost several Hox genes, and thus the Hox code must be simpler in modern tardigrades then it was in the last common ancestor of Panarthropoda [75]. Tardigrades are also missing a *dachshund* ortholog, indicating that leg patterning is simpler in modern tardigrades than it was in the last common ancestor of Panarthropoda [82]. In this study, we discovered that tardigrades have lost several Wnt genes. Other miniaturized animals are also missing several Wnt genes. In fact, Tardigrada, Nematoda, and Rotifera, which are all miniaturized animals [73, 74, 91, 92], exhibit the highest level of gene loss among animals with sequenced genomes [105]. In terms of the body plan, miniaturization is associated with anatomical simplification and reduction in cell number [73, 74, 91, 92]. Development of the simple body plans of highly miniaturized animals would not be expected to require the complex mechanisms that control development of larger animals. As developmental mechanisms, such as posterior growth or cell fate specification mechanisms that require complex combinatorial interactions, are lost in association with miniaturization, the genes that once regulated these processes may also be lost. Therefore, we propose that independent cases of extreme miniaturization in animals explain remarkable examples of convergence in terms of genome and developmental evolution.

## Methods

### Identifying candidate genes and phylogenetic analyses

Reciprocal BLAST searches were performed to identify candidate genes. We collected sequences from a genome assembly for *R. varieornatus* [85], and a genome assembly [71], an embryonic transcriptome assembly [126], and an adult transcriptome assembly [127] for *H. exemplaris*. We also collected sequences from genome or transcriptome assemblies for *A. vaga* [94], *S. mediterranea* [95], *H. miamia* [96], *D. gyrociliatus* [97], and *E. kanangrensis* [65]. All other sequences that we included in our phylogenetic analyses were publicly available in GenBank. We confirmed that candidate genes encoded predicted protein domains by CD search analysis [83]. For phylogenetic analyses, protein sequences were aligned with MUSCLE [128]. For Wnt analyses, we aligned sequences to a previously published matrix [3]. Information about sequences used in phylogenetic analyses is available in Additional file 4: S2. Alignments were trimmed using Gblocks [129, 130]. Alignments were visually inspected in Mesquite [131]. Alignments are available in Additional file 5: FASTA alignments. The LG model [132] was used for phylogenetic analyses, with an estimated proportion of invariable sites and an estimated gamma shape parameter with four substitution rate categories. Maximum likelihood analyses were performed with PhyML [133]; branch support was calculated by bootstrap (500 replicates) and the aLRT SH-like method. We produced majority rule consensus trees in Mesquite with required tree topologies set to 0.5 from three maximum likelihood trees [131]. Bayesian analyses were performed with MrBayes with Nchains = 4 [134]. Tracer was used to diagnose convergence [135]. Posterior probabilities were calculated from 4500 trees from the posterior tree distribution.

### PCR and cloning

Primers were designed from *H. exemplaris* gene sequences (sequences available upon request). GoTaq Green Master Mix (Promega) was used to amplify fragments of Wnt genes from *H. exemplaris* embryonic cDNA. Fragments were cloned into the pCR4-TOPO TA vector (Invitrogen). This strategy worked for all Wnt genes besides *He-Wnt16A* and *He-WntA*.* He-Wnt16A* was amplified from a single exon from genomic template. A *He-WntA* fragment was synthesized by Integrated DNA Technologies. Sanger sequencing was performed by Eton Bioscience to confirm the identity of cloned or synthesized sequences.

### In situ hybridization and imaging

In situ hybridization was performed by following a published protocol [136]. This protocol has been used successfully for several previous studies of tardigrade development [75, 76, 82]. After completion of the in situ hybridization protocol, embryos were mounted on slides in DAPI-Flouromount-G (SouthernBiotech). DIC and fluorescence images were captured on an Olympus FV1000 Fluoview confocal microscope. We used the fluorescence properties of the chromogenic in situ hybridization stain to capture confocal data [137]. Fluorescence data were collected using a UPlanSApo 100×/1.40 oil objective, a 405 nm laser to capture DAPI data, and a 635 nm laser using the Cy5 excitation and detection presets in the Olympus Fluoview software to capture in situ data. Brightness and contrast of confocal stacks were adjusted in ImageJ. Images were produced in the Volume Viewer plugin in ImageJ. Image levels were adjusted in Photoshop.

## Supplementary Information

**Additional file 1: Figure S1.** Majority rule consensus tree of Wnt ligands. This tree is the same tree shown in Fig. 1, but includes branch length information. See Fig. 1 caption for additional details.

**Additional file 2: S1.** Results of reciprocal BLAST searches for orthologs of Arrow/ LRP5/LRP6. *D. melanogaster* Arrow sequence was used to query protein databases. The top ten matches were analyzed by reciprocal BLAST search against *D. melanogaster* reference proteins. The source of the Subject sequences is provided. For Subject sequences that are not available in GenBank, we provide a link to the source of the data, the ID of each sequence in the data set from which it was recovered, and each sequence.

**Additional file 3: Figure S2.** In situ hybridization results for Wnt genes that were expressed across entire embryos, were very weakly expressed, or exhibited inconsistent expression patterns at one or more developmental stages in *H. exemplaris*. All images are DIC micrographs. Purple staining represents gene expression. Anterior is towards the top. **a–c** *Wnt2* expression was weak at all stages investigated except for stage 11. **d** *Wnt4* expression was weak during stage 14. **e–h** *Wnt9* was broadly expressed during all stages investigated, but may have been more highly expressed in the endomesodermal layer. **e–g** Left panels are ½ probe concentration. **i–l** *Wnt11* expression was detected throughout the embryo but was strongest at the posteriormost region. **m–p** *Wnt16A* expression was weak or undetectable as all stages investigated.

**Additional file 4: S2.** Sources of sequences in phylogenetic analyses in this study. For sequences that are not available in GenBank, we provide a link to the source of the data, the ID of each sequence in the data set from which it was recovered, and each sequence. For the Wnt analyses, all sequences besides those from tardigrades and *Euperipatoides kanangrensis* were part of a previously published matrix [3]. GenBank Accession numbers for tardigrade Wnt and Frizzled sequences are available in Table 1.

**Additional file 5: FASTA alignments.** Alignments used in phylogenetics analyses in this study.

## Data Availability

All sequence data analyzed in this study are publicly available. *H. exemplaris* sequence data are from NCBI BioProject PRJNA360553 [71]. *R. varieornatus* sequence data are from NCBI BioProject PRJDB4588 [85]. GenBank accession numbers for other sequences that we analyzed are available in Additional file 2: S1 and Additional file 4: S2. Links to data sources and sequences are available in Additional file 2: S1 and Additional file 4: S2 in cases where sequences do not have GenBank accession numbers.

## Abbreviations

Ad: *Adineta vaga*
ant: Anterior
Ap: *Acyrthosiphon pisum*
AP: Anteroposterior
APC: *Adenomatous polyposis coli tumor suppressor*
*arm*: *Armadillo*
cDNA: Complementary deoxyribonucleic acid
Ce: *Caenorhabditis elegans*
Cg: *Crassostrea gigas*
Ct: *Capitella teleta*
Cs: *Cupiennius salei*
cWnt: Canonical Wnt
DAPI: 4′: 6-Diamidine-2′-phenylindole dihydrochloride
Dg: *Dimorphilus gyrociliatus*
DIC: Differential interference contrast
*Dll*: *Distal-less*
Dm: *Drosophila melanogaster*
Dp: *Daphnia pulex*
EGF: Epidermal growth factor
*en*: *Engrailed*
Ek: *Euperipatoides kanangrensis*
ep1–ep4: Endomesodermal pouch 1–endomesodermal pouch 4
fg: Foregut
Fz: Frizzled
Gm: *Glomeris marginata*
*He*: *Hypsibius exemplaris*
*hh*: *Hedgehog*
Hm: *Hofstenia miamia*
Hs: *Homo sapiens*
Is: *Ixodes scapularis*
l1–l4: Leg 1–leg 4
Lrp: Low-density lipoprotein receptor
Mm: *Mus musculus*
nt: Nucleotide
Ob: *Octopus bimaculoides*
*pan*: *Pangolin*
PCR: Polymerase chain reaction
Pd: *Platynereis dumerilii*
PD: Proximodistal
pos: Posterior
Pt: *Parasteatoda tepidariorum*
*Rv*: *Ramazzotius varieornatus*
*sgg*: *Shaggy*
Sm: *Schmidtea mediterranea*
t1–t4: Trunk segment 1–trunk segment 4
Tc: *Tribolium castaneum*
*wg*: *Wingless*
Xl: *Xenopus laevis*
Zn: *Zootermopsis nevadensis*
