# Supplementary material for: Extensive loss of Wnt genes in Tardigrada
Source: BMC Ecol Evol. 2021 Dec 27;21:223. doi: 10.1186/s12862-021-01954-y (PMC8711157; doi:10.1186/s12862-021-01954-y)

Extensive loss of Wnt genes may be related to miniaturization in Tardigrada

Authors:
Raul A. Chavarria; Mandy Game; Briana Arbelaez; Chloe Ramnarine; Zachary K. Snow; Frank W. Smith*

Additional file 1_Fig_S1

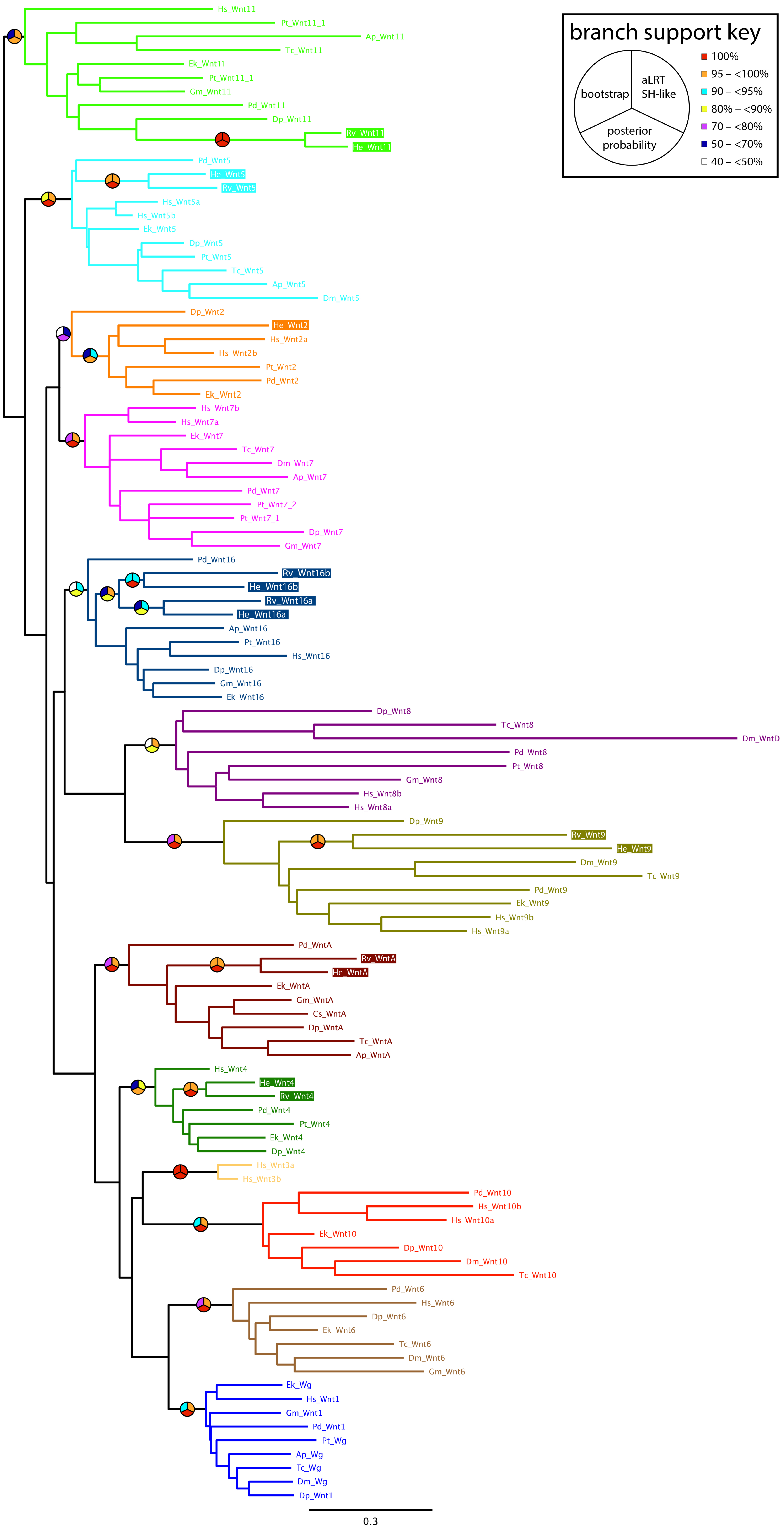

Supplement: Supplementary file 1 — Additional file 1: Figure S1. Majority rule consensus tree of Wnt ligands. This tree is the same tree shown in Fig. 1, but includes branch length information. See Fig. 1 caption for additional details. [file 12862_2021_1954_MOESM1_ESM.pdf]
