# Supplementary material for: Extensive loss of Wnt genes in Tardigrada
Source: BMC Ecol Evol. 2021 Dec 27;21:223. doi: 10.1186/s12862-021-01954-y (PMC8711157; doi:10.1186/s12862-021-01954-y)

Extensive loss of Wnt genes may be related to miniaturization in Tardigrada

Authors:
Raul A. Chavarria; Mandy Game; Briana Arbelaez; Chloe Ramnarine; Zachary K. Snow; Frank W. Smith

Additional file 3_Fig_S2

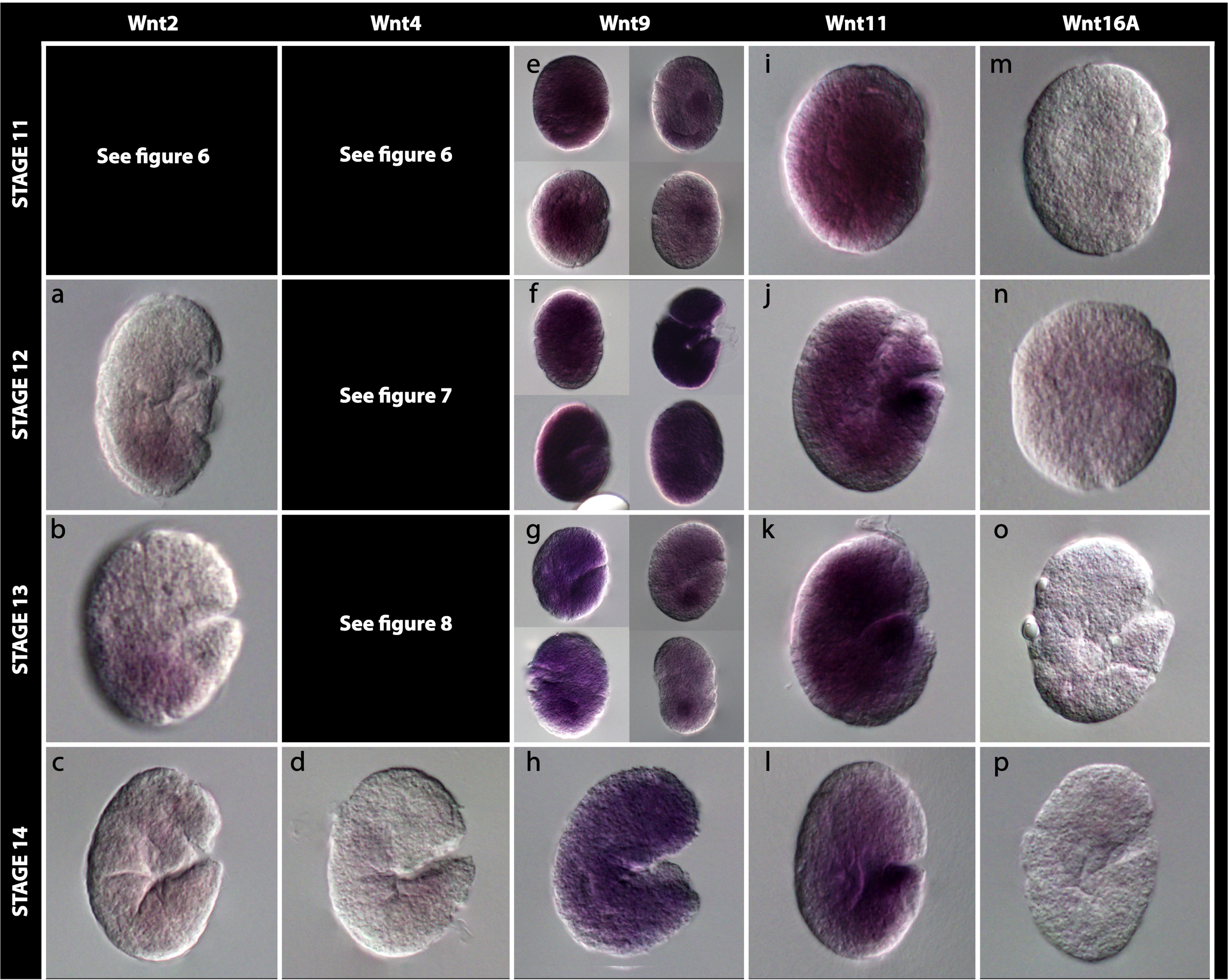

Supplement: Supplementary file 3 — Additional file 3: Figure S2. In situ hybridization results for Wnt genes that were expressed across entire embryos, were very weakly expressed, or exhibited inconsistent expression patterns at one or more developmental stages in H. exemplaris. All images are DIC micrographs. Purple staining represents gene expression. Anterior is towards the top. a–c Wnt2 expression was weak at all stages investigated except for stage 11. d Wnt4 expression was weak during stage 14. e–h Wnt9 was broadly expressed during all stages investigated, but may have been more highly expressed in the endomesodermal layer. e–g Left panels are ½ probe concentration. i–l Wnt11 expression was detected throughout the embryo but was strongest at the posteriormost region. m–p Wnt16A expression was weak or undetectable as all stages investigated. [file 12862_2021_1954_MOESM3_ESM.pdf]
